# A Shocking Complication: Right Ventricular Pacemaker Lead Perforation Presenting With Intractable Hiccups

**DOI:** 10.7759/cureus.93262

**Published:** 2025-09-26

**Authors:** Jeremy M Williams, Melville C O'Brien, Pramod Reddy

**Affiliations:** 1 Internal Medicine, University of Florida College of Medicine - Jacksonville, Jacksonville, USA

**Keywords:** cardiac device lead migration, ct (computed tomography) imaging, intractable hiccups, pacemaker lead complication, right ventricular lead perforation

## Abstract

Right ventricular (RV) lead perforation is a rare but potentially life-threatening complication after pacemaker implantation, with clinical presentations ranging from asymptomatic to severe hemodynamic compromise. Early diagnosis is challenging due to the heterogeneity of symptoms and overlap with other conditions. We present a unique case of a 60-year-old man who presented with intractable hiccups two days after dual-chamber pacemaker implantation. Initial workup was unrevealing; however, persistent symptoms prompted further review of imaging, which revealed RV lead perforation with migration into the left anterior chest wall. The patient underwent successful extraction and repositioning of the lead, resulting in the resolution of symptoms. This case highlights the diagnostic challenge posed by atypical presentations of RV lead perforation, such as persistent hiccups resulting from phrenic nerve or diaphragmatic irritation. Computed tomography is the most sensitive non-invasive diagnostic tool, but a high index of suspicion is essential for timely diagnosis and management. Early intervention is critical to prevent severe complications.

## Introduction

Pacemaker implantation is a common intervention used in the management of various cardiac arrhythmias [1]. While generally considered a benign procedure, it does carry the risk of complications such as right ventricular (RV) lead perforation. The etiology of RV lead perforation is thought to be multifactorial, with a combination of mechanical factors and patient-related characteristics [2]. The clinical presentation of RV lead perforation can vary widely, ranging from being asymptomatic to requiring intensive care unit (ICU) level of care if hemodynamic instability is present [3]. The vague symptomatology, along with similarities to a variety of other conditions, makes early diagnosis of this complication challenging. There is no universally accepted "gold standard test" to diagnose RV lead perforation; however, computed tomography (CT) is widely considered the best diagnostic tool available as it demonstrates a sensitivity of 97% and allows for a direct visualization of cardiac structures [4]. While the decision to obtain CT imaging depends on provider discretion and requires a high level of suspicion, physicians must recognize the potential symptoms of this condition and act swiftly in order to reduce morbidity. We highlight an atypical presentation of RV lead perforation in a patient with a chief complaint of intractable hiccups and initial imaging that proved to be falsely reassuring.

## Case presentation

A 60-year-old man with a past medical history of sick sinus syndrome and recent placement of a dual-chamber pacemaker five days prior presented to the hospital with a chief complaint of intractable hiccups starting roughly two days after pacemaker implantation. The hiccups were episodic, lasting several hours at a time, and associated with a vague left-sided chest pain and subjective shortness of breath. The pain was described as pleuritic with worsening upon deep inspiration and radiating to the left side of his abdomen. He denied any recent trauma, palpitations, nausea, vomiting, diarrhea, fevers, pacemaker site tenderness, or any other inciting event prior to symptom onset. Furthermore, he endorsed compliance with his sling use and refrained from making any overhead movements, as previously instructed. 

**Figure 1:**
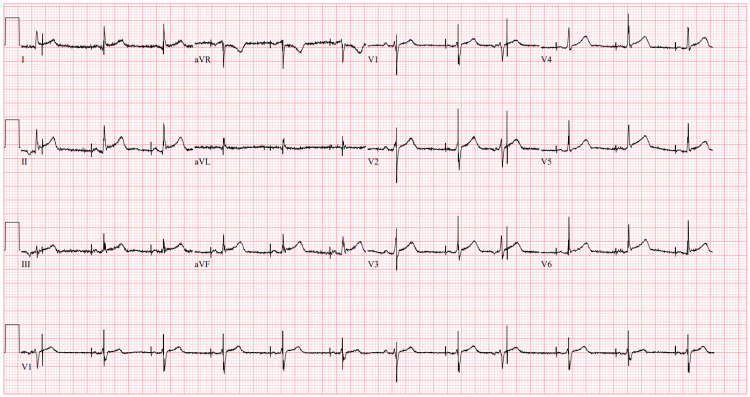
Initial ECG Upon Arrival to the ED Twelve-lead ECG demonstrating AV dual-paced rhythm with a rate of 70 beats per minute (BPM) and no sign of acute ischemic changes.

**Figure 2:**
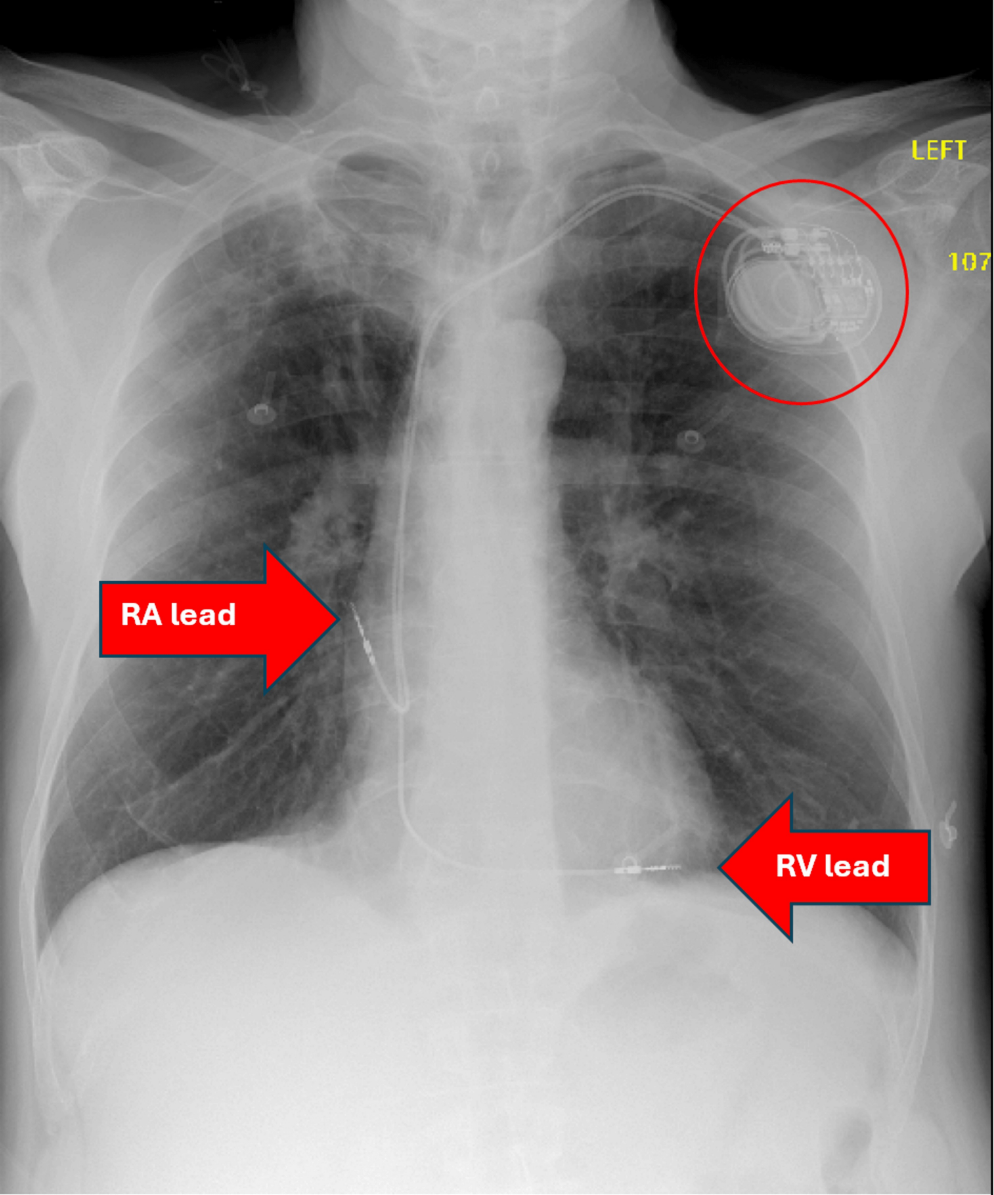
Initial Chest X-Ray Plain chest radiography showing pacemaker device with distal lead tips seemingly in appropriate position within the right atrium (RA) and right ventricle (RV). No obvious pericardial effusion or focal consolidation noted.

**Figure 3:**
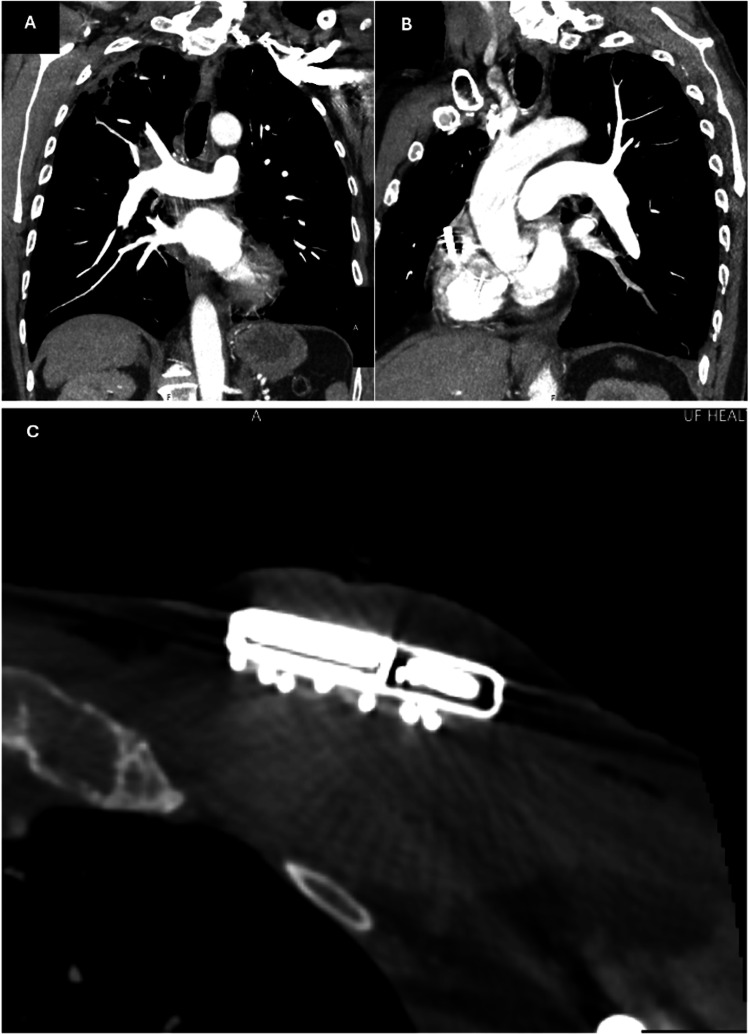
Computed Tomography Angiogram (A,B) CT angiogram, coronal view, demonstrating a lack of acute thrombus within the right and left pulmonary system, respectively. (C) CT angiogram, transverse view, demonstrating a lack of soft tissue edema or fat stranding.

Vital signs on presentation were significant for an oxygen saturation of 94% on two-liter nasal canula with a respiratory rate of 29 respirations per minute; blood pressure and heart rate were within normal limits. On physical exam, there were no areas of apparent chest wall trauma, and the pacemaker insertion site was without swelling, erythema, purulent discharge, or tenderness to palpation. Mild bilateral wheezing was noted on pulmonary auscultation; however, cardiac examination was grossly unremarkable with no audible rubs, murmurs, or gallops. Laboratory findings were notable for a white blood cell (WBC) count of 12.4×10^3^/uL, with negative high-sensitivity cardiac troponin, erythrocyte sedimentation rate (ESR), and C-reactive protein (CRP) levels. Electrocardiogram (ECG) showed an atrioventricular (AV) dual-paced rhythm with a rate of 70 beats per minute (BPM) and no sign of acute ischemic changes (Figure 1). Chest X-ray showed a normal-sized cardiac silhouette, both pacemaker leads seemingly in appropriate position, and no findings of focal consolidation (Figure 2). The patient was administered a gastrointestinal (GI) cocktail for suspected gastroesophageal reflux disease (GERD); however, this did not provide relief. He was then put on empiric treatment for chronic obstructive pulmonary disease (COPD) exacerbation and given muscle relaxers for suspected post-operative musculoskeletal pain. Given the clinical context, the decision was subsequently made to obtain computed tomography angiography (CTA) of the chest, which was negative for acute pulmonary embolism (PE) or any areas of localized soft tissue inflammatory changes that might indicate a pocket infection around the pacemaker (Figure 3).

**Figure 4:**
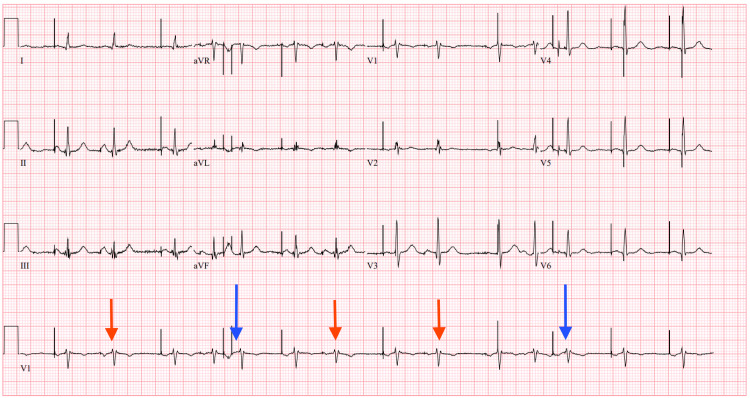
Subsequent ECG Concerning for Pacemaker Malfunction Twelve-lead ECG on day 2 of admission showing atrial-paced rhythm with frequent sinus complexes (red arrows) and occasional premature atrial contraction (PACs; blue arrows) concerning for pacemaker malfunction.

**Figure 5:**
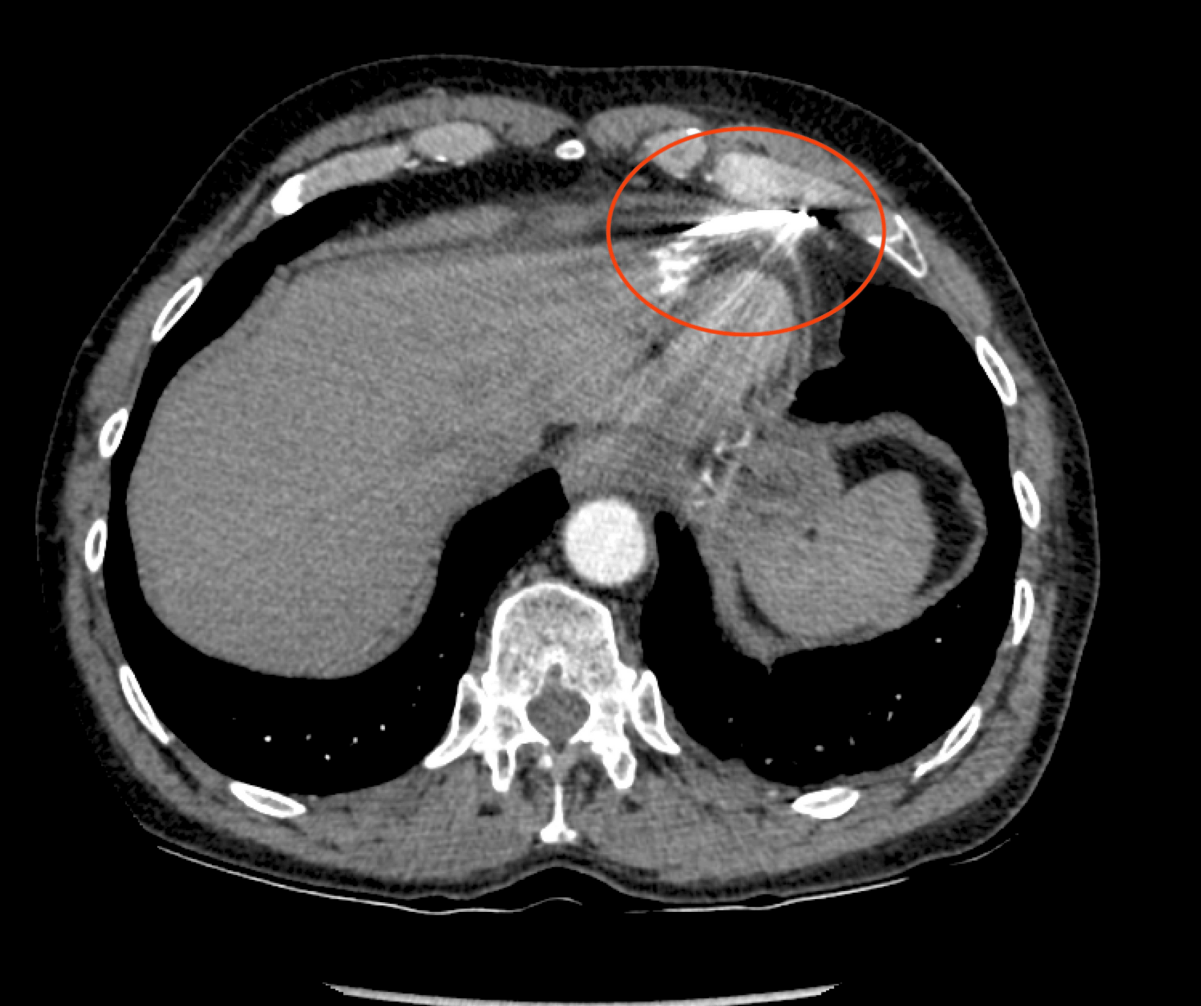
Pacemaker Lead Perforating Right Ventricle (RV) Found on Image Review 2-D computed tomography demonstrating RV lead perforation with the distal lead tip (red circle) within the anterior chest wall.

**Figure 6:**
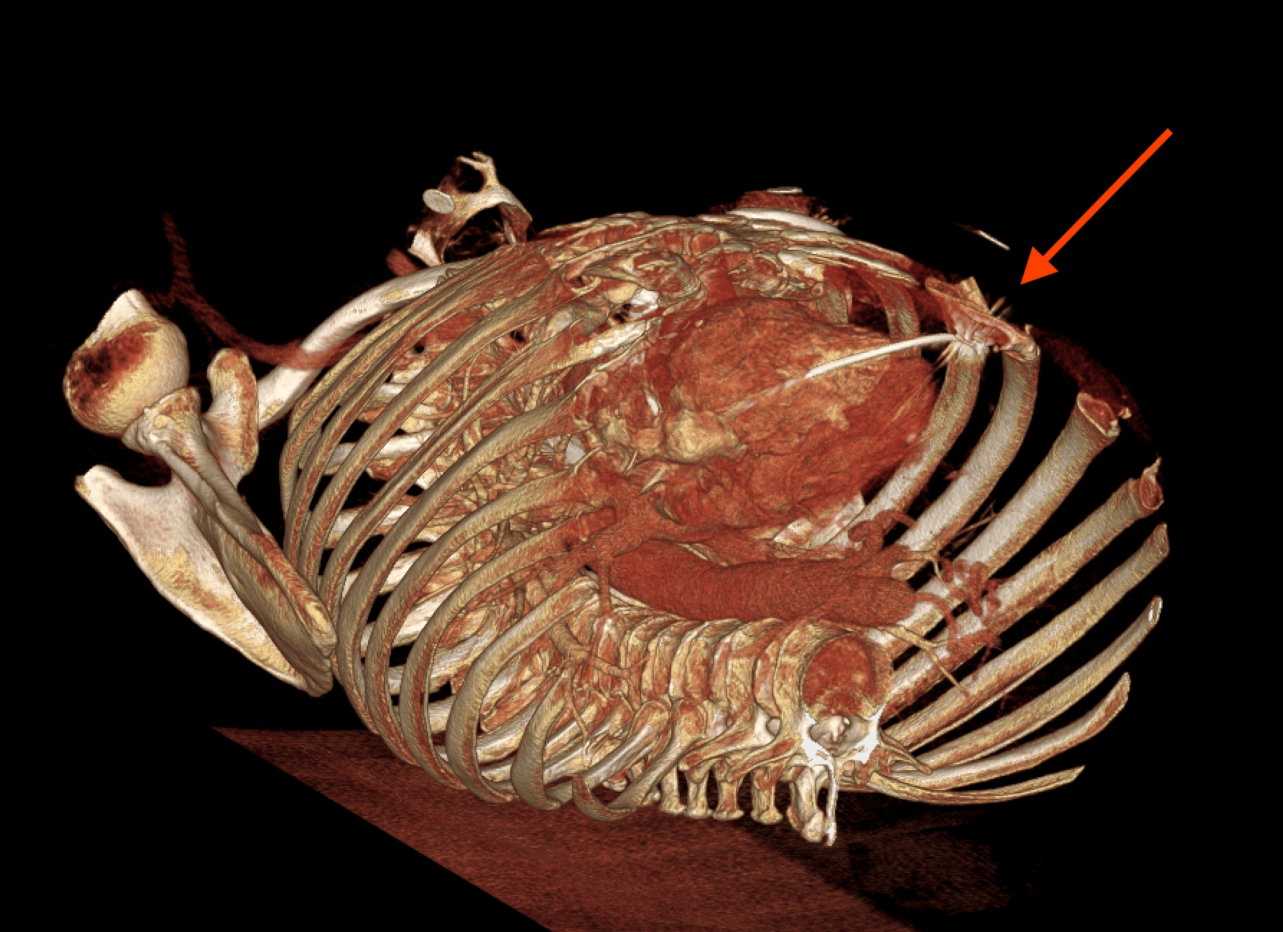
Three-Dimensional Visual of Right Ventricular (RV) Lead Perforation 3-D reconstruction from chest CT imaging, showing a caudo-cranial view of RV lead perforation. Red arrow pointing to distal lead tip within the anterior chest wall after puncturing through RV wall. Image created using Visage Imaging Software (Visage 7 platform, version 2.5.0; Visage Imaging, Inc., San Diego, CA, USA) by Dr. Jeremy Williams.

Despite treatment for COPD exacerbation and optimization of multi-modal pain control, the patient's symptoms showed minimal improvement. A repeat ECG on day 2 of hospitalization again showed an atrial-paced rhythm, however, now with frequent sinus complexes and occasional premature atrial contractions (PACs), concerning for pacemaker malfunction (Figure 4). The electrophysiology (EP) team performed a bedside device interrogation without findings of notable arrhythmogenic events. Given the lack of improvement, the primary team re-visited the patient's imaging and noticed a possible pacemaker lead perforation that was not noted on the original exam findings (Figures 5, 6). The radiology team was consulted and confirmed the patient's distal pacemaker lead had perforated the RV wall and was embedded within the left anterior chest wall. An urgent transthoracic echocardiogram was performed, revealing a small pericardial effusion without any signs of tamponade or pre-tamponade physiology. The patient was then taken to the EP lab, where the extraction and repositioning of the RV lead was successfully performed under fluoroscopic guidance. The patient tolerated the procedure well and remained hemodynamically stable throughout the remainder of the hospitalization with no acute arrhythmogenic events on telemetry monitoring. He reported near-complete resolution of symptoms and was eventually discharged with continued improvement noted on an outpatient EP follow-up one week later.

## Discussion

This case highlights how challenging the diagnosis of RV lead perforation can be, as the clinical manifestations vary widely from asymptomatic to life-threatening emergencies. The mimicry of other non-cardiac conditions can easily mislead physicians and prompt a workup for a broad range of differential diagnoses. The unique aspect of this case is the highly atypical presentation for RV lead perforation, manifesting primarily as intractable hiccups rather than stabbing chest pain or hemodynamic compromise. Furthermore, the difficulty of diagnosis was amplified by falsely reassuring findings on initial imaging, underscoring the importance of maintaining a high level of clinical suspicion and exercising sound clinical judgment.

RV lead perforation is a known but uncommon complication of pacemaker insertion. Although infrequent, with large multicenter studies reporting an incidence of approximately 0.5% among over 10,000 implants [5], this complication can be severe and potentially fatal. The most common presenting symptom is pleuritic chest pain occurring in nearly half of all cases; however, patients may also present with symptoms such as shortness of breath, syncope, dizziness, or be completely asymptomatic [6]. Frequent or constant hiccupping, as reported by our patient, is an uncommon but recognized symptom that can occur when a displaced lead stimulates the diaphragm or phrenic nerve [3]. Although this symptom is atypical, it should raise clinical suspicion for possible RV lead perforation, especially in the context of recent pacemaker placement. 

Early recognition and accurate identification of RV lead perforation are vital for appropriate management and for preventing severe complications. While pericardial effusion and cardiac tamponade associated with lead perforation are less common, they can lead to significant morbidity and mortality [7]. Since presentations can mimic more acute conditions such as PE or acute coronary syndrome (ACS), it is appropriate to rule these conditions out as part of the workup. ECG abnormalities are observed in the majority of cases and may be the first indication of lead perforation [8]. Point-of-care ultrasound (POCUS) is valuable for rapid bedside assessment, especially in unstable patients, but may not always clearly show lead tip location. Imaging plays a crucial role in detecting lead perforation as CT demonstrates high sensitivity (97%) for this condition, while chest X-rays and echocardiography are also commonly used to detect lead migration or any associated effusions [4]. 

While unclear in this case, the etiology for RV lead perforation can vary and is often multifactorial. Mechanically, the use of active fixation leads, improper technique, or excessive lead slack during implantation can increase the risk of perforation [2]. Furthermore, patient risk factors that may predispose to perforation include older age, female sex, low body mass index, and chronic corticosteroid use, all of which may correlate with thinner myocardial walls and increased tissue fragility [2,9]. Management of patients with RV lead perforation depends on the clinical presentation and severity of the condition. Asymptomatic or incidentally discovered chronic perforations may not require any intervention; however, those with life-threatening complications, such as cardiac tamponade or hemodynamic instability, may necessitate urgent interventions, including emergency pericardiocentesis or immediate surgical repair [10]. The majority of patients are managed successfully with image-guided lead extraction and repositioning [3,11].

## Conclusions

RV lead perforation, though uncommon, can present with highly atypical symptoms that mimic other conditions, making early diagnosis difficult. Persistent or unusual symptoms following pacemaker implantation, such as intractable hiccups, should prompt consideration of lead perforation, even when initial imaging is inconclusive. Computed tomography remains the most sensitive diagnostic modality, but clinical vigilance is paramount. Early recognition and prompt management, typically with image-guided lead extraction and repositioning, are essential to reduce morbidity and improve patient outcomes.

## References

[1] Mulpuru S, Madhavan M, McLeod C, Cha YM, Friedman PA (2017). Cardiac pacemakers: function, troubleshooting, and management: part 1 of a 2-part series. J Am Coll Cardiol.

[2] Satomi N, Enta K, Otsuka M, Ishii Y, Asano R, Sawa S (2021). Left ventricular free wall perforation by a right ventricular pacemaker lead: a case report. Eur Heart J Case Rep.

[3] Akbarzadeh MA, Mollazadeh R, Sefidbakht S, Shahrzad S, Bahrololoumi Bafruee N (2017). Identification and management of right ventricular perforation using pacemaker and cardioverter-defibrillator leads: a case series and mini review. J Arrhythm.

[4] Rajkumar CA, Claridge S, Jackson T (2017). Diagnosis and management of iatrogenic cardiac perforation caused by pacemaker and defibrillator leads. Europace.

[5] Waddingham PH, Elliott J, Bates A (2022). Iatrogenic cardiac perforation due to pacemaker and defibrillator leads: a contemporary multicentre experience. Europace.

[6] Kumar P, Skrabal J, Frasure SE, Pourmand A (2022). Pacemaker lead related myocardial perforation. Am J Emerg Med.

[7] Deshpande S, Swatari H, Ahmed R (2023). Predictors of morbidity and in-hospital mortality following procedure-related cardiac tamponade. J Arrhythm.

[8] Son J, Jung LY (2023). Unusual presentation of asymptomatic subacute lead-related ventricular perforation beyond the pericardium without pericardial effusion: a case report. J Yeungnam Med Sci.

[9] Rodgers JL, Jones J, Bolleddu SI (2019). Cardiovascular risks associated with gender and aging. J Cardiovasc Dev Dis.

[10] Ebrahimi P, Taheri H, Bahiraie P (2025). Incidence of secondary pericardial effusions associated with different etiologies: a comprehensive review of literature. J Cardiothorac Surg.

[11] Mori H, Kato R, Ikeda Y (2020). Percutaneous simple lead traction is a feasible and effective method for right ventricular lead perforations. Int Heart J.

